# A structural insight into the negative effects of opioids in analgesia by modulating the TLR4 signaling: An *in silico* approach

**DOI:** 10.1038/srep39271

**Published:** 2016-12-16

**Authors:** Masaud Shah, Muhammad Ayaz Anwar, Dhanusha Yesudhas, Jayalakshmi Krishnan, Sangdun Choi

**Affiliations:** 1 Department of Molecular Science and Technology, Ajou University, Suwon, 443-749, Korea; 2 Department of Life Sciences, Central University of Tamil Nadu, Neelakudi, Thiruvarur, Tamil Nadu, India

## Abstract

Opioids are considered the gold standard therapy for pain. However, TLR-dependent negative effects in analgesia have highlighted the complexities in the pharmacodynamics of opioids. While successive studies have reported that morphine and Morphine-3-glucuronide (M3G) activate the TLR4 pathway, the structural details of this mechanism are lacking. Here, we have utilized various computational tools to reveal the structural dynamics of the opioid-bound TLR4/MD2 complex, and have proposed a potential TLR4 activation mechanism. Our results support previous findings, and include the novel insight that the stable binding of morphine and naloxone, but not M3G, in the MD2 cavity, is TLR4 dependent. Morphine interacts with MD2 near its Phe126 loop to induce the active conformation (MD2^C^); however, this binding is likely reversible, and the complex gains stability upon interaction with TLR4. M3G also induces the MD2^C^ state, with both the Phe126 loop and the H1 loop being involved in MD2-M3G complex stability. Remarkably, naloxone, which requires TLR4 interaction for complex stability, switches the conformation of the gating loop to the inactive state (MD2°). Cumulatively, our findings suggest that ligand binding and receptor clustering occur successively in opioid-induced TLR4 signaling, and that MD2 plasticity and pocket hydrophobicity are crucial for the recognition and accommodation of ligands.

The association between pain and immunity was discovered decades ago, when it was revealed that interleukin-1β (IL-1β) was involved in the induction of sickness-induced hyperalgesia and hyperplasia *per se*^1^,^2^. Soluble mediators, released by injured tissues and immune cells in the central nervous system (CNS), bind to the pre- and post-synaptic terminals and toll-like receptors (TLRs) on microglial and endothelial cells^3^,^4^. This activates complex signaling processes that intersect at multiple points, modulating inhibitory and excitatory synaptic processes and thereby resulting in allodynia and nociceptive hypersensitivity. Among the TLRs, TLR2 and TLR4 have been widely reported to be associated with gliosis in studies of the neuroimmune interface^5^,^6^,^7^.

Opioids, considered the benchmark therapy for both chronic and acute pain, also have drawbacks, such as the potential for abuse, opioid-induced tolerance, and an association with paradoxical hyperplasia. While such disadvantages are partly associated with neuronal factors, the involvement of opioids in immune signaling pathways affects the body’s ability to modulate opioid metabolism^8^. The stimulation of central immune signaling events following TLR4 activation has underlying consequences for opioid pharmacodynamics, including an impairment of the efficacy of opioids as analgesics, leading to hyperalgesia. The sterioselective activity of morphine and its metabolites is vital for classical opioid receptor signaling. Morphine is glucuronidated in liver cells to form M3G and M6G. M3G has no opioid activity, and its long-term use can oppose analgesia and boost nociception^8^,^9^,^10^, whereas M6G actively binds to opioid receptors, and thus exhibits analgesic potential^11^,^12^. TLR4 can be activated by both the (−)-morphine and (+)-morphine isomers, only the first of which can also activate the opioid receptor^13^.

Multiple *in vitro, in vivo*, and *in silico* models have been used to investigate the role of morphine, and its metabolite M3G, in TLR4 signaling. Hutchinson *et al*. 2009, utilized an *in silico* to *in vitro* prediction model, as well as a separate *in silico* docking model, and predicted that morphine, and its opioid-inactive metabolite M3G, specifically bind to the lipopolysaccharide (LPS)-binding pocket of MD2 rather than to TLR4^14^. Successive studies have expanded on these findings to suggest that the activation of the TLR4 pathway by morphine and M3G can be non-stereoselectively blocked by (+/−)-naloxone; this was further supported by *in silico* model^10^. Both *in vivo* and biophysical assays have suggested that the binding of morphine to MD2 facilitates the oligomerization of TLR4 and triggers proinflammatory responses. Additionally, disruption of the TLR4/MD2 interaction was found to abolish morphine-induced inflammation *in vitro*, and to potentiate morphine analgesia *in vivo*^15^. A summary of *in silico* studies investigating possible morphine, M3G, and naloxone modulation of TLR4, as well as their possible binding interfaces on MD2, is presented in Table 1 5,10,13,14,15,16.

Structural studies, such as X-ray crystallography and NMR, can reveal the precise orientation of components of protein-protein and protein-ligand complexes. However, as well as being extremely laborious, these techniques provide limited information about time-dependent structural dynamics. Scientists have therefore developed comprehensive computational techniques such as molecular dynamics simulation (MDS), based on Newtonian physics, which predicts the movement of atoms within proteins over time^17^. MDS and other *in silico* techniques have been employed to validate experimental data and to provide structural insights into TLR4/MD2 and MD2-ligand interactions^18^,^19^,^20^. The interaction of morphine, M3G, and naloxone with TLR4/MD2 has been validated by preliminary *in silico* analyses, but the stability and accuracy of these models are limited. However, the structural details of the interactions of these ligands with the TLR4/MD2 complex or with MD2 alone, as well as the detailed structural changes in these complexes that are likely to govern the (in)activation of the TLR4 pathway, are yet to be investigated. Comprehensive computational methods are required for the prediction of possible binding poses of these ligands with the TLR4/MD2 complex, and for the prediction of structural details that could be used to modulate opioid-induced hyperalgesia and nociception. This study focuses on the structural dynamics of opioid-bound TLR4/MD2, as well as on possible mechanisms for the non-stereoselective activation by morphine and M3G or inhibition of the TLR4 pathway by naloxone.

## Results

### *In silico* docking simulations

**Figure 1:**
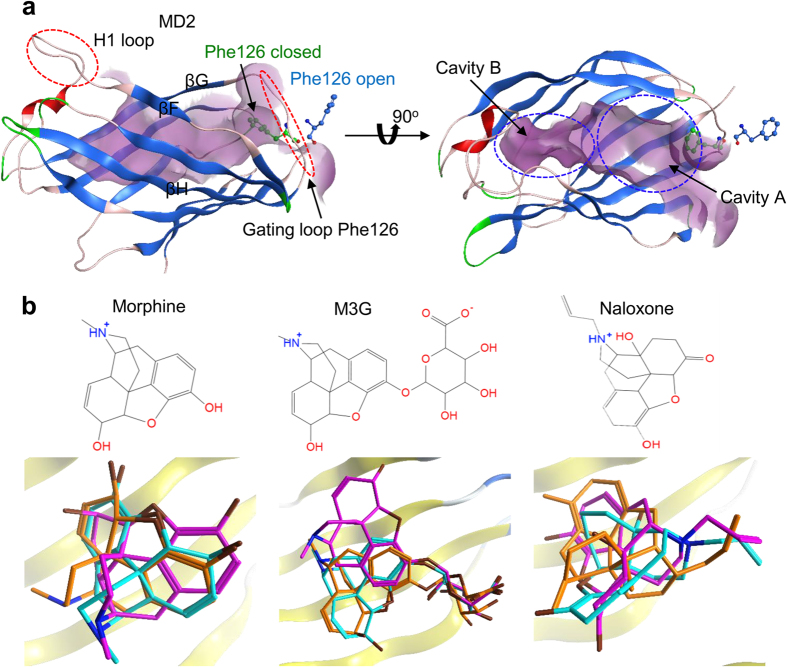
Opioids and the structural features of MD2 in its active (MD2^C^) and inactive (MD2°) states. (**a**) The two loops, H1 and Phe126 (red dotted circles), are crucial for MD2-TLR4 dimerization and TLR4-TLR4 oligomerization, respectively. The orientation of the Phe126 sidechain within the gating loop is important for receptor clustering after ligand binding. If the loop is in the closed conformation, MD2 is productive, and induces TLR4 activation. Conversely, the open conformation induces the non-productive MD2 state and halts TLR4 signaling. (**b**) Depiction of the structures of morphine, M3G, and naloxone. The lower panel shows the superimposition of the top scoring MD2-ligand complexes, determined using the three docking tools MOE (pink), AutoDock (orange) and PatchDock (cyan). Morphine is bound exclusively in cavity A, while the main scaffold of M3G binds within cavity A and its glucuronide moiety binds in cavity B.

Several studies, which aimed to dock opioids and other compounds with MD2 and the TLR4/MD2 complex *in silico*, considered the LPS-binding pocket as the only available ligand-binding cavity (Table 1). However, LPS is a huge molecule that interacts, both electrostatically and hydrophobically, with more than 35 of the 158 amino acid residues in MD2. We studied morphine, M3G and naloxone, which are comparatively less bulky than LPS and thus occupy a smaller proportion of the MD2 ligand-binding pocket. MD2 is an extremely flexible molecule that can accommodate ligands of various sizes by adjusting its cavity volume accordingly^18^. The MD2 binding cavity can be divided into two cavities, A and B (Fig. 1a). Cavity B is near the H1 loop (loop between βF and βG strands) and is deep enough to be inaccessible to solvent molecules; however, cavity A is bounded by the gating loop and is solvent-accessible. It was recently reported that morphine and M3G are bound within the cavity of MD2, but also interact allosterically with other parts of both the dimeric and tetrameric forms of the TLR4/MD2 complex^21^. However, these findings contradict other studies reviewed in Table 1, which suggest that these ligands are preferentially bound within the cavity of MD2, with no allosteric binding to the TLR4/MD2 complex. To investigate these inconsistencies and suggest a possible binding pocket, the docked complexes of morphine, naloxone, and M3G with the active form of MD2 (MD2^C^), both alone and as part of the TLR4/MD2^C^ heterodimer, were simulated and investigated. All three of the docking tools used directed these ligands into the MD2^C^ pocket, with defined poses, occupying cavities A or B. The docking tools used allowed us to visualize the superposed docking poses of morphine and naloxone in cavity A, and of M3G in cavity B (Fig. 1b). The stability of the most conformant docking pose, determined by the molecular operating environment (MOE) docking score (listed in Table 2), was further investigated.

#### Morphine

**Figure 2:**
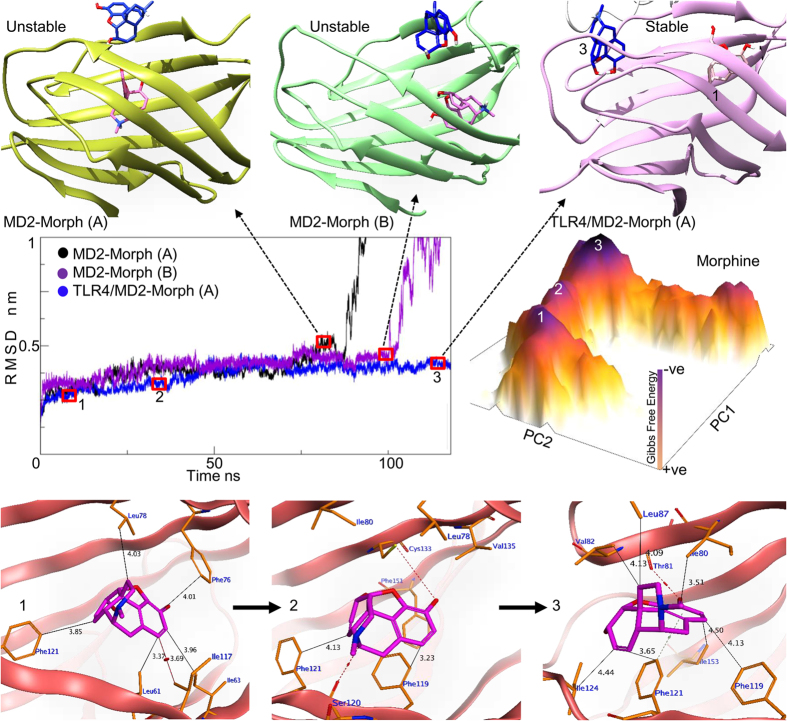
Morphine-binding affinity and structural dynamics of MD2. Morphine binds weakly to MD2 in the absence of TLR4 (top yellow and green cartoons). These complexes dissociated after 80 ns and 100 ns of molecular dynamics simulation (MDS) respectively, as shown in the RMSD plot of the backbone atoms of MD2 and morphine (Black and purple plots). In the presence of TLR4, morphine maintains its contacts with MD2 and transitions from the initial binding position 1 to the final position 3 (pink cartoon, top right). The plateau represents the free energy landscape (FEL) of the MD2-morphine complex during MDS (dark purple peaks represent the lowest energy states). The numbers on each peak correspond to the RMSD time step (blue) at which the system was in its most stable, lowest energy state. The orange cartoons at the base of the figure represent the protein-ligand interfaces at particular low energy states. Phe121 and Ile124 are vital for the movement of morphine from its initial binding pocket to the Phe126 loop. A and B represent the cavities in which the ligand was docked.

Morphine, when docked with uncomplexed MD2^C^ alone, displayed predominantly hydrophobic interactions with both the solvent-accessible cavity A and the buried cavity B, with differing interacting residues being involved in different poses (Table 2). However, all of the MD2^C^-morphine interactions, irrespective of docking pose or binding cavity, were lost during MDS. However, when morphine was docked with TLR4/MD2^C^ heterodimer, the complex retained its stability throughout the simulations. When in a stable binding conformation in the MD2^C^ cavity, morphine binds near to the Phe126 residue of the Phe126 loop (From here on, “Phe126 loop” refers to the entire loop between the βG and βH strands, while “Phe126” refers to the single phenylalanine residue at position 126), primarily through hydrophobic interactions with Phe119, Phe121, and Ile124 (Fig. 2). This suggests that TLR4 may be a stabilizing factor for the MD2^C^-morphine complex. To investigate this further, the allosteric effect of TLR4 on MD2^C^-morphine binding was monitored over time by observing the root-mean-square fluctuation (RMSF) of the MD2^C^ residues in both the monomeric and heterodimeric (TLR4/MD2^C^) forms. The residues Cys95-Ser102 displayed a dramatically lower RMSF in the heterodimeric TLR4/MD2^C^ form than in the monomeric MD2^C^ form (Supplementary Fig. S1). Further, β-factors indicated that the atoms of the residues in this loop vibrate considerably more vigorously in the MD2^C^ monomer than in the TLR4/MD2^C^ heterodimer (Supplementary Fig. S2).

#### M3G

Docking simulations of M3G with either TLR4/MD2^C^ or MD2^C^ showed that the metabolite occupied a similar region of the MD2^C^ cavity A to morphine. M3G established hydrogen bonding interactions with Tyr102 and hydrophobic interactions with hydrophobic residues, in particular Leu78 and Phe151. However, the interactions in cavity A were not resilient and the complexes dissociated during MDS. This could be due to the hydrophilic glucuronide group of M3G, which is important for the MD2^C^-M3G interaction, being readily solvent-accessible in cavity A. In contrast to morphine, which binds near the gating loop within cavity A, M3G exhibited stable binding throughout MDS near to the H1 loop of cavity B. β-factor measurements indicated that residues in the H1 loop, in addition to those of the Phe126 loop, vibrate markedly, which may be vital for MD2^C^-M3G complex stability. The stability of the binding of M3G to MD2^C^ in cavity B was investigated by calculating the distance between M3G and Ser103 over time using MDS, and could be largely attributed to electrostatic interactions (Supplementary Fig. S3). Of the three ligands that were investigated, M3G showed the highest binding affinity for MD2^C^ (−8.6 kcal/mol), consistent with previous biophysical and *in silico* studies^21^. The stability of the MD2^C^-M3G complex was associated with the formation of hydrogen bonds between two −OH groups and a −COOH group of the glucuronide moiety of M3G, and the polar Ser103 in H1 loop and Thr115 in cavity B, respectively (Table 2). The interaction of the main scaffold of M3G with cavity A overlaps with that of morphine and naloxone, and establishes additional hydrophobic contacts for stability.

#### Naloxone

The docking of naloxone was investigated using the same parameters as for morphine and M3G, and, although it bound to both cavities A and B, the whole MD2^C^-naloxone complex was unstable and dissociated during MDS. As with morphine, the complex of naloxone docked with the TLR4/MD2^C^ dimer was stable. However, unlike the MD2^C^-morphine complex, which exhibited substantial fluctuation in multiple loop regions, the MD2^C^-naloxone complex showed considerably less fluctuation overall, with the Phe126 loop being the exception (Supplementary Fig. S1). The overall stability of the complex was considerably increased by the addition of TLR4. β-factor measurements for MD2^C^ complexed with naloxone, but not with the agonistic morphine and M3G, indicated that, in addition to Phe126, the residues Ile124 and Lys125 exhibited high vibration levels.

Our initial docking results suggest that morphine binds within cavity A of MD2^C^, and that this interaction is stabilized by the presence of TLR4. M3G, a metabolite of morphine that does not activate the opioid receptor, establishes stable electrostatic interactions within cavity B and interacts with part of cavity A via hydrophobic bonds, irrespective of the presence or absence of TLR4. Like morphine, naloxone requires TLR4 to stably interact with MD2^C^, and fits neatly into cavity A. The docking data for morphine, M3G, or naloxone into cavity B of MD2^C^ complexed with TLR4 is not shown here. However, supplementary data has been provided showing the docking conformation of morphine and naloxone in TLR4/MD2 complex (Supplementary Fig. S4).

### Structural dynamics of the MD2^C^-morphine complex

Biophysical, *in vitro*, and exploratory *in silico* analyses have confirmed the interaction of morphine with MD2 (Table 1). However, in the absence of a definitive structure of the complex, computational tools are able to present plausible data concerning the binding patterns, stability, dynamic changes, and domain movements of the MD2-morphine complex, as well as hypotheses to explain the mechanisms by which TLR4 antagonizes the analgesic properties of morphine.

The highest scoring pose for morphine bound within cavity A of MD2^C^ was selected for molecular dynamics analysis. When bound initially, morphine places itself deep within the cavity, where its =N-CH3 group forms arene-H bond with the hydrophobic sidechain of Phe151. To understand the stability of the MD2^C^-morphine complex, whole complex was subjected to MDS under CHARMM force field. As shown in the root-mean-square deviation (RMSD) graph in Fig. 2, the complex was unstable and dissociated after 80 ns. When the simulation procedure was replicated using three different poses of morphine within the cavity A, the complex dissociated at different time steps. This suggests two potential causes of complex instability; firstly, that morphine binds to other region within the MD2^C^ cavity, or, secondly, that MD2 needs TLR4 in order to maintain this interaction for a significant length of time. To investigate further, a separate morphine-binding pose, this time within cavity B of MD2, was subjected to MDS using the same parameters. This binding interaction also dissociated unexpectedly after 100 ns (Fig. 2). The structural dynamics of the TLR4/MD2^C^-morphine complex were then investigated using the same protocol as above. As expected, this complex retained its stability throughout the simulation run-time, suggesting that while morphine initially interacts with soluble MD2, subsequent association with TLR4 is required to maintain this interaction and to induce downstream signaling.

The residues of MD2 residing in the binding pocket play key roles in ligand binding and recognition, and the two loops, Phe126 and H1, are likely to be involved in both the MD2-TLR4 interaction and distinguishing the (ant)agonistic functions of binding ligands. Our findings suggest that these loops are directly involved in MD2-morphine stability, which is partly governed by the presence of TLR4. A comparative RMSF plot indicates that the presence of TLR4 enhances MD2^C^-morphine complex stability through the stabilization of the Cys95-Ser102 loop region (Supplementary Fig. S1), which is supported by lower β-factor values from the TLR4/MD2^C^-morphine complex (Supplementary Fig. S2). The molecular dynamics of these loops were compared to those of intact MD2^C^ to establish their role in the TLR4/ MD2^C^-morphine complex. The RMSD graph for this analysis shows that the H1 loop remained stable during the simulation, whereas the Phe126 loop deviated from its initial conformation. Further, the RMSF graph also indicates that the Phe126 loop residues showed higher fluctuation than other parts of the protein (Supplementary Fig. S5a).

### Morphine-bound MD2 retains its active conformation (MD2^C^) during MDS

Principal component analysis (PCA) is widely used to examine the principal movements related to events during MD simulation, represented as eigenvalues and eigenvectors (EVs), exhibited by the domains of a bimolecular complex. The calculated eigenvalues and eigenvectors allow the motion of molecules during a specific molecular event to be examined in depth.

Cartesian coordinate PCA (cPCA) was performed for the complete MD2 protein, using the Cartesian coordinates of the backbone atoms, to determine the dominant movements within the MD2^C^-morphine complex. A total of ten EVs were extracted from the 120 ns simulation trajectory, and their contribution to the total fluctuation of MD2 was examined. The first two principal components (PCs) were used to give an overall impression of the free-energy landscape (FEL), and revealed one dominant native state and two metastable states (Fig. 2). The structural evolution of the complex over time was then predicted using structural coordinates from these low energy states. This analysis suggested that morphine initially binds deep within cavity A, but then moves towards the Phe126 loop over time, establishing hydrophobic contacts with the Phe121 and Ile124 residues (Fig. 2). It is clear that Phe121 plays an important role in the movement of morphine towards the Phe126 loop. We investigated this further by monitoring the distance between Phe121 and morphine, and, interestingly, we observed a clear pulling trend, with the initial 5 Å distance being reduced by half after 25 ns (Supplementary Fig. S3). In addition to the contacts with the Phe126 loop described above, the −OH moiety of morphine forms a hydrogen bond with the backbone C=O of Thr81, and also interacts with the residues Ile80, Val82, Leu87, Phe119, and Ile153. Interestingly, all of the interfacial residues listed above reportedly interact with the synthetic TLR4 agonist neoseptin-3^22^, consistent with both our *in silico* docking model and the suggested binding pose of morphine in TLR4-bound MD2^C^.

**Figure 3:**
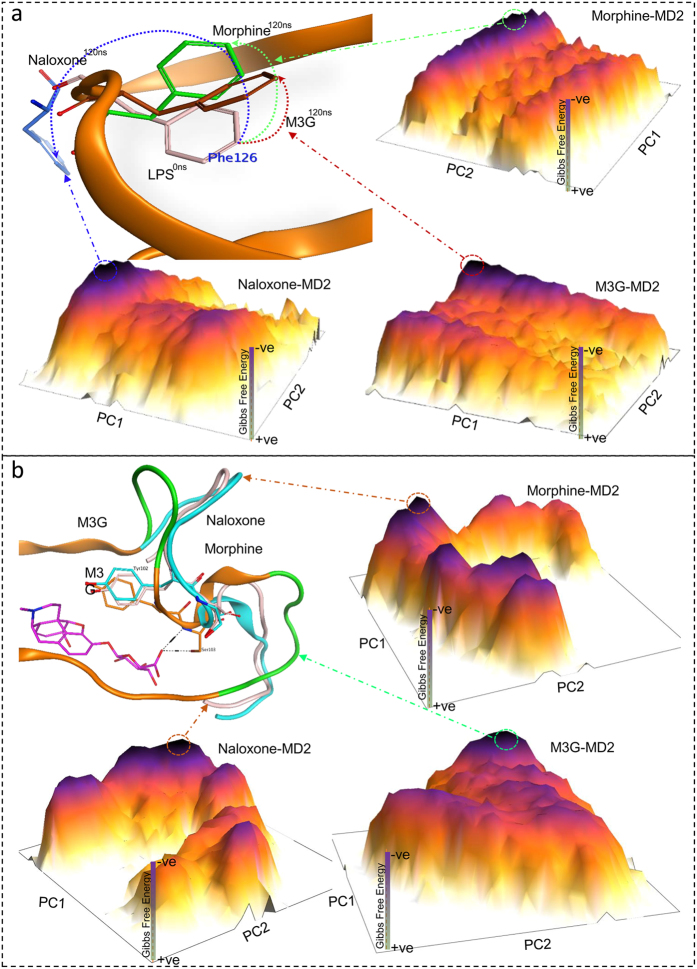
Dihedral principal component analysis (dPCA) of the Phe126 loop in ligand-bound MD2. (**a**) The sidechain rotation of Phe126 explains the agonistic or antagonistic nature of the bound ligands. The position of Phe126 in the LPS-bound MD2^C^ crystal structure is shown. The degree of Phe126 rotation was defined as the difference between the initial and final structural coordinates, sampled from the lowest energy native states (the peaks in FEL plateaus) following dPCA. In naloxone-bound MD2, the Phe126 sidechain shifts markedly from a closed to open conformation, conversely, it remains within the MD2 pocket when bound to either morphine or M3G. In the cartoon representation, the brown, green, and blue dotted arrows indicate the displacement of Phe126 in MD2 complexed with M3G, morphine, or naloxone, respectively. (**b**) dPCA of the H1 loop supports the investment of this loop in MD2-M3G stability. Tyr102 and Ser103 remain stable in this loop and interacts with M3G, however, the two green parts (Cys95-Asp100 and Ala107-Val113) of this loop fluctuate vigorously as compared to morphine and naloxone-bound MD2.

As discussed above, the Phe126 loop of MD2 is crucial for the (in)activation of TLR4, and its residues showed high fluctuation in an RMSF plot. The FEL calculated with cPCA is slightly diffuse, and few conformational states could be identified, possibly because of the mixing of internal motion with overall motion. To clarify the (ant)agonist switching movement of the Phe126 loop, dihedral principal component (dPCA) analysis was performed on the loop’s backbone dihedral angles. The FEL from this analysis clearly predicted that the sidechain of the Phe126 residue remains inside the MD2 pocket over time, maintaining the closed MD2^C^ conformation (Fig. 3a).

Moreover, *in silico* dynamics analysis predicts that morphine binds to soluble MD2 in the vicinity of the gating loop, to promote the agonistic conformation. This interaction is reversible and is unstable in the absence of TLR4, but gains stability when TLR4 is bound to MD2, even though there is no direct interaction between morphine and TLR4.

### Structural dynamics of the MD2^C^-M3G complex

Similar to morphine, the TLR4-agonistic properties of M3G have been studied, and its interaction with MD2, as well as dimeric and tetrameric TLR4/MD2, has been explored using biophysical and preliminary *in silico* methods. We utilized MDS in order to provide deeper insights into the interaction mechanisms of these bimolecular complexes. As reported previously, the MD2 ligands display three main binding modes: binding within the mouth of MD2, binding an area spanning the mouth to the base of the cavity of MD2, and covalent binding^19^. Considering this and the highest scoring poses of M3G docked within defined cavities of MD2 were selected for simulation analysis.

**Figure 4:**
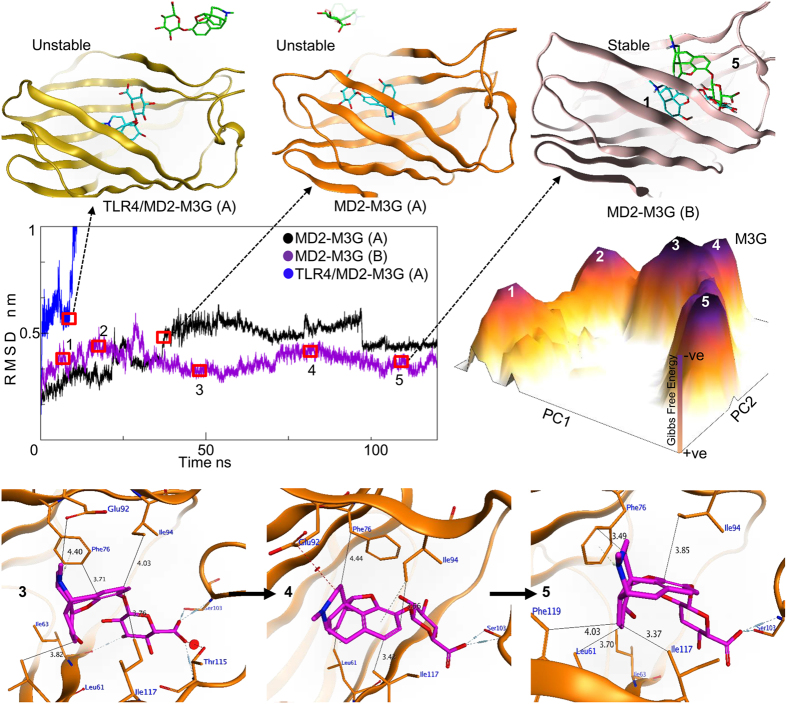
M3G-binding affinity and an investigation of the structural dynamics of MD2. The stability of the MD2-M3G complex when ligand is bound within sub-cavities A and B is not affected by the presence of TLR4. When M3G is docked within cavity A, it dissociates in both the presence and absence of TLR4 (top orange and yellow cartoons), whereas the complex is stable, irrespective of TLR4 binding, when the ligand is docked within cavity B. Structural dynamics analysis suggests that M3G loses contact with MD2 at an early stage when bound within cavity A (black and blue RMSD plots), but remains in complex with MD2 when bound within cavity B (purple plot). The plateau represents the free energy landscape (FEL) (dark purple peaks represent the lowest energy states of the MD2-M3G complex). The number on each peak corresponds to the RMSD time step at which the system was at its lowest energy state (purple plot). The orange cartoons at the base of the figure depict the protein-ligand interfaces at particular low energy states. A and B denote the cavities in which the ligand was docked.

Docking simulation data from this and previous studies^21^ show that M3G binds more readily and with higher affinity to MD2 than morphine, possibly through its glucuronide group. Most MD2 ligands form hydrophobic bonds deep within its cavity, while ligand’s phosphate groups interact electrostatically with the positively charged residues at the mouth of MD2^19^. Similar to the procedure followed with morphine, we investigated three M3G poses within cavity A, in which the glucuronide group was oriented in different directions. In all of the simulated MD2^C^-M3G complexes, M3G bound close to the mouth of MD2^C^, and dissociated after differing lengths of time (Fig. 4, showing data for a single pose). We therefore observed the effect of TLR4 on the MD2^C^-M3G complex by simulating the TLR4/ MD2^C^-M3G complex, with M3G bound within cavity A, using the same duration (120 ns) and parameters as previously stated. Surprisingly, unlike with MD2^C^-morphine, TLR4 had no positive effect on the stability of the MD2^C^-M3G complex. This suggested that M3G may have another stable binding orientation within the MD2^C^ cavity, and so a docked MD2^C^-M3G complex, in which the negatively charged glucuronide group was bound near to the H1 loop, deep within cavity B, was investigated. M3G binds deeper within the MD2^C^ cavity than morphine^21^, in a location where the R3 finger of LPS has been shown by X-ray crystallography to form hydrophobic bonds^23^. This H1 loop has been investigated in the crystallographic studies to participate and stabilize the TLR4/MD2 dimer interface^23^. Additionally, *Garate and Oostenbrink* recently studied the opening and closing events of TLR4-bound MD2 in the absence of ligand, speculating that the H1 loop increases MD2 ligand-binding capacity^19^, while a TLR4 inhibitor reportedly bound to the H1 loop binding interface with TLR4 to block the MD2-TLR4 interaction. Deciphering the dynamics of this loop could explain the (ant)agonistic behavior of MD2-binding ligands.

The RMSD graph shown in Fig. 4 indicates that the MD2^C^-M3G complex was initially fluctuating, but was stable from the 30 ns time step until the end of the simulation (Fig. 4). In addition to the Phe126 loop, M3G-bound MD2^C^ also exhibited substantial fluctuation in H1 loop (Supplementary Fig. 5b), suggesting that, unlike morphine, which binds near the Phe126 loop, M3G binds deep within the MD2^C^ cavity and affects the conformation of both the Phe126 and H1 loops. Comparative RMSF analysis suggests that the presence of TLR4 reduces the fluctuation in the H1 loop (Supplementary Fig. 1). However, MD2^C^-ligand stability seems to depend on the binding pose and electrostatic interactions of M3G in the MD2^C^ cavity. To investigate the H1 loop contacts further, the distance between Ser103 and M3G was measured for the duration of MD simulation. Within cavity A, M3G was displaced from its initial position irrespective of the presence of TLR4, although in the TLR4/MD2^C^-M3G heterodimer, the displaced M3G still interacted allosterically with TLR4. This allosteric interaction was however not seen consistently when the process was repeated (data not shown). Conversely, when M3G was bound within cavity B, a remarkably stable interaction was maintained through hydrogen bonding between Ser103 and M3G (Supplementary Fig. 3). Our dynamics results suggest that, unlike the interaction with morphine, where the H1 loop made no significant contribution, when M3G was bound to MD2^C^ both the H1 and Phe126 loops vibrated substantially (Supplementary Fig. 5c). The high β-factor values for these loops when M3G is bound, unlike when morphine is bound, support this observation (Supplementary Fig. 3). The investment of loop H1 in MD2-M3G complex was further investigated through dPCA of the backbone dihedral angle of this loop. The PDB coordinates sampled from the lowest energy native state of the FEL graph indicate that this loop remained dormant in the presence of morphine and naloxone, but bent upward when MD2 was bound to M3G. However, two residues, Tyr102 and Ser103, did not change its sidechain positions (Fig. 3b). Ser103 in particular displayed a crucial role in MD2-M3G stability, as discussed above.

### M3G retains the active conformation of MD2^C^ irrespective of the presence of TLR4

As described above for the MD2^C^-morphine complex, cPCA was used to investigate structural changes when MD2^C^ interacts with M3G. The FEL was plotted using the first two principal components, PC1 and PC2, and the energy barriers and structural changes exhibited by the complex throughout the simulation were noted. The peaks in the FEL represent the lowest Gibbs energy states, and the plateau indicates that the complex remained in two native states for most of the simulation, although multiple metastable states can also be seen (Fig. 4). Structural coordinates representing the lowest energy peaks in the FEL were sampled from the MD trajectory, and indicate that the MD2^C^-M3G complex evolves over time before attaining its native state at the 100 ns time step (state 5 in the FEL plot, Fig. 4). Detailed interface analysis suggests that the main M3G scaffold interacts with the same MD2^C^ interface as morphine, however, its glucuronide group forms hydrogen bonds with the polar Ser103 and Thr115 residues, located near the H1 loop. As the simulation progressed, the −O atom of the carboxylic moiety of the glucuronide group lost its interaction with Thr115. However, M3G retained its interaction with Ser103, as well as most of its hydrophobic interactions, for the entire duration of the simulation (Supplementary Fig. 3). As seen previously with morphine, a FEL drawn using dPCA of the backbone dihedral angles of the Phe126 loop suggested that the Phe126 sidechain retained a closed conformation (Fig. 3a).

These results suggest that the main scaffolds of M3G and morphine initially select the same MD2^C^ binding pocket, but the presence or absence of the charged glucuronide moiety is critical for the establishment of the lower energy native conformation. Furthermore, the structural dynamics of the MD2^C^-M3G complex suggest that, in addition to the Phe126 loop, the H1 loop is potentially important for ligand-binding affinity and the overall stability of the TLR4/ MD2^C^-M3G complex.

### Structural dynamics of MD2^C^ when bound to naloxone

**Figure 5:**
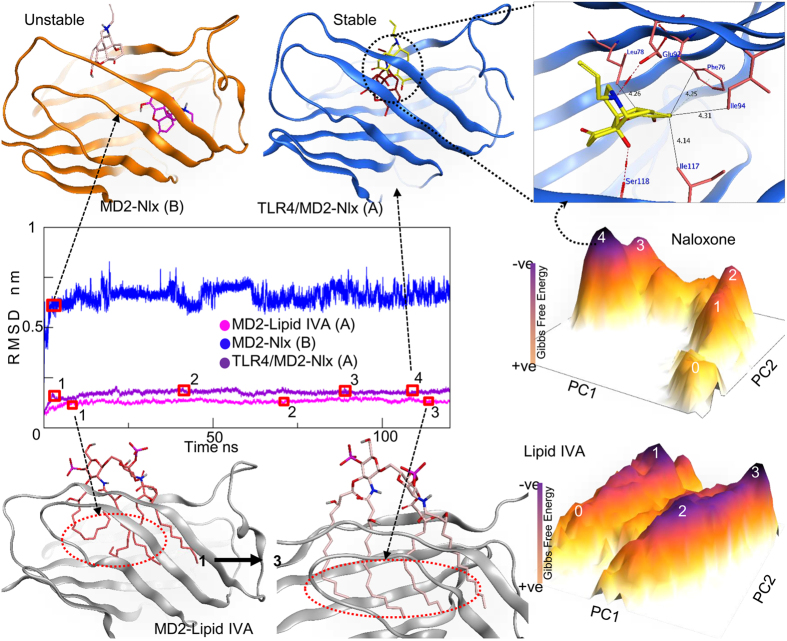
Structural dynamics of MD2 bound to the TLR4 antagonists naloxone and lipid IVa. TLR4 receptor clustering is key in MD2-naloxone complex stability. In the absence of TLR4, naloxone dissociates from MD2 early in MDS (RMSD, blue line), irrespective of the binding cavity used (top left, orange cartoon). Conversely, in the presence of TLR4, the MD2-naloxone complex is stable, as is the MD2-lipid IVa complex (purple and pink plots, respectively). The plateaus represent the FEL, with dark purple peaks indicating the lowest energy states of the MD2-ligand complex. The number on each peak corresponds to the RMSD time step (purple, MD2-naloxone and pink, MD2-lipid IVa) at which the systems were in their lowest energy states. The blue cartoons at the top right of the figure represent the MD2-naloxone interface in native state 4 from the FEL plateau. A and B denote the cavities in which the ligand was docked.

Multiple *in vitro* studies have reported that naloxone can both impair opioid analgesia and counteract TLR4-dependent hyperalgesia and allodynia (Table 1). Additionally, both (+)- and (−)-naloxone have been shown to bind to the opioid-binding pocket of TLR4/MD2 and opioid receptors, halting their signaling. We investigated this computationally, and proposed an antagonistic TLR4 effect. Like morphine, naloxone depends on TLR4 to stabilize its interaction with MD2 (Fig. 5). The main scaffolds of naloxone and morphine are similar, and the two ligands bind to the same cavity region, in particular through hydrophobic interactions with Phe76, Leu78, and Ile117, in TLR4-bound MD2. However, while morphine moves to the Phe126 loop during MDS, naloxone retains its initial binding position throughout the MDS. This enhanced stability can be attributed to the two hydrogen bonds established at the mouth of the MD2^C^ cavity, between the −OH and C-N groups of naloxone and the backbone C=O of Ser118 and sidechain −COOH group of Glu92, respectively. This suggests that naloxone could competitively block the entry of morphine, M3G, and LPS into the cavity of MD2, and subsequently hamper TLR4 signaling. The structural dynamics of MD2-bound LPS and lipid IVa were in line with our previously reported structural dynamics study and we did not proceed for detail investigation^18^,^24^. However, the stability of MD2-lipid IVa during MDS has been depicted in Fig. 5. The structural coordinates for native state 3, extracted from the FEL plot, suggest that the acyl chains of lipid IVa relax and extent deeper into the MD2 cavity that is seen in the native crystal complex (Fig. 5).

### Naloxone spontaneously switches the active MD2^C^ conformation into inactive MD2° form

The overall structural dynamics of MD2-naloxone and MD2-morphine in heterodimeric case, TLR4/MD2, showed quite similar trends (Supplementary Fig. 5a,d), however, the FELs for MD2^C^ and the Phe126 loop, generated by cPCA and dPCA, respectively, differed. The FEL plot suggested a single native state for MD2^C^-naloxone in the TLR4/MD2^C^-naloxone complex, although several metastable states were also observed in the FEL plateau (Fig. 5). Structural coordinates extracted from the single native state indicate that the Phe126 sidechain spontaneously swivels through 180°, thereby switching the active closed state (MD2^C^) into an inactive open state (MD2°), potentially explaining how naloxone negatively regulates TLR4 (Fig. 3a). This finding is supported by comparative β-factor analysis, which suggests that, unlike morphine and M3G, naloxone greatly influences the stability of the entire Phe126 loop, in particular the Lys122, Lys125, Phe126, and Lys128 residues, explaining why conformation switching is seen only in the MD2^C^-naloxone complex.

In summary, our findings show that the location of naloxone binding within the MD2^C^ pocket is similar to that of morphine, lipid IVa, LPS, and the recently reported TLR4 agonist, Neoseptin-3. Remarkably, naloxone switches the gating loop conformation within MD2 from the active (MD2^C^) to the inactive (MD2°) form, although TLR4 is required for further stabilization. This suggests that naloxone is unlikely to block the TLR4/MD2 interaction, but instead abolishes TLR4 signaling via another mechanism. We propose that naloxone induces the non-productive (MD2°) conformation of MD2 in a similar manner to lipid IVa, and competes with morphine and other TLR4 agonists.

### Plasticity of MD2 is important for ligand binding and the modulation of TLR4 signaling

**Figure 6:**
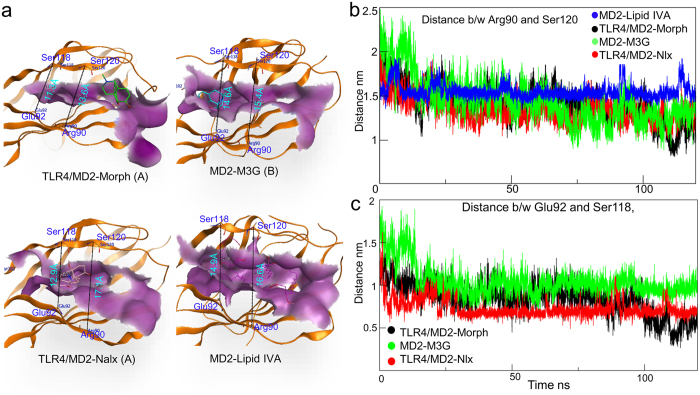
Cavity dynamics in ligand-bound MD2. (**a**) The cavity of MD2 shrinks to fit to the size of the bound ligand. The distance between charged residues located at the MD2 cavity mouth reduced during simulations of morphine or M3G-bound MD2, naloxone-bound MD2 and remained unchanged in case of lipid IVa-bound MD2. (**b**) The distance between Arg90 and Ser120 was investigated for the full 120 ns simulation trajectory. (**c**) The distance between Glu92 and Ser118 was investigated for the full 120 ns simulation trajectory. A correlation was found between the size of the ligand and the distance measured, suggesting the plasticity of MD2 is vital for optimal ligand interactions. A and B denote the cavities in which the ligand was docked. Morph; morphine; Nlx: naloxone.

The plasticity and hydrophobicity of MD2 have been widely studied in the context of ligand accommodation and hydrophobic collapse. Consistent with our results, it was previously reported that the cavity of MD2 is remarkably malleable in both the open and closed states, and undergoes a “clamshell-like” motion to adjust its volume and precisely match the ligand’s proportions^18^. While hydrophobic collapse and reduction in MD2 cavity volume were not considered in this study, a remarkable reduction in the main hydrophobic patch area within the MD2 cavity was found using a protein patch analysis protocol (part of the MOE software suite) (Table 2). The initial cavity area of the MD2^C^ form was almost the same for all docking simulations, at approximately 11.6% of the total MD2 protein area. During MDS however, the hydrophobic patch within the cavity reduced to half the initial area (Table 2). In order to track the distinctive clamshell-like motion of MD2, we monitored the distance between the Cαs of the Arg90 and Ser120 residues present at the mouth of the MD2 cavity. This distance is 17.72 Å in the TLR4/MD2-LPS crystal structure (3FXI), but this gap is reduced by 4.1 Å to 13.62 Å when morphine is bound (Fig. 6a). Binding of M3G and naloxone to MD2^C^ also reduced this gap, although to a lesser extent than morphine, to 15.4 Å and 17.1 Å respectively. To investigate this gap reduction in the gating residues, and shrinkage of the MD2 cavity, the distances between the Cα of the residues listed above and Glu92 and Ser118 were evaluated for all of the systems throughout the simulation. As expected, a prominent, gradual reduction of this gap was seen whenever MD2 was bound to morphine, M3G, or naloxone (Fig. 6b,c), although, interestingly, no plasticity of the cavity was seen when lipid IVa was bound to MD2. Similar results have been reported previously, suggesting that the reduction in the MD2 cavity volume is related to ligand size^18^.

### Hydrophobicity of the MD2 pocket is vital for interaction with ligand

The amino acid residues in the drug-binding pocket of a protein are critical for ligand selection and the maintenance of protein-ligand complex stability. MD2 has only one ligand-binding pocket, composed mainly of hydrophobic residues. The contribution that the pocket residues make towards ligand affinity and complex stability varies with the size and properties of the ligand, which is clearly seen in crystal structures of MD2 bound to ligands of various sizes^22^,^23^,^25^. Alanine-scanning mutagenesis is often used to rapidly identify amino acids with crucial roles in protein structure, function and stability^26^,^27^. Substituting a residue of choice with alanine is a good strategy, because it neither changes the main-chain conformation nor imposes any significant electrostatic effects^28^.

**Figure 7:**
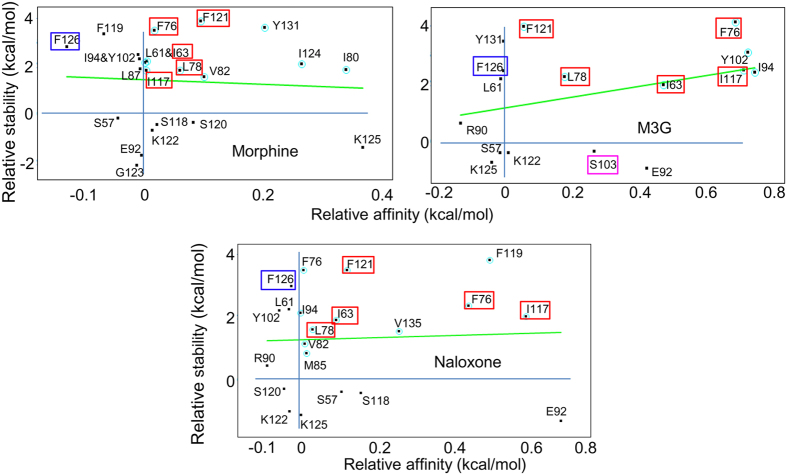
Combinatorial alanine scanning of the ligand-bound MD2 cavity. Alanine scanning was used to determine the relative contributions of MD2 pocket residues to ligand affinity and complex stability. Phe126 (blue box) does not directly participate in ligand binding, but destabilizes the MD2-ligand complex when mutated, suggesting an allosteric influence on the complex. The red boxes represent the hydrophobic residues within the MD2 cavity that are directly involved in ligand binding and complex stabilization. Contacts with these residues were made by all three ligands, although mutation of other, unshared residues could also influence complex stability. Ser103 (pink box) directly interacts with M3G through hydrogen bonds, and stabilizes the complex.

We implemented the same strategy to decipher the contributions of MD2 pocket residues to ligand binding, and their relative impact on mutant complex stability. Structural coordinates for MD2-morphine, MD2-M3G, and MD2-naloxone, obtained by using the FEL plateau to determine the lowest energy native state, were used in computational alanine scanning, wherein key MD2 cavity residues, as determined by point mutation experiments^29^,^30^,^31^ and our docking study, were mutated. When the selected pocket residues were mutated, drastic changes in both MD2 stability and ligand-binding affinity were observed (Fig. 7). In addition to the deleterious effects on ligand-binding affinity and complex stability, seen with mutation of ligand-bound residues, we noted a significant allosteric effect on MD2-ligand stability when more distant residues were mutated. Five hydrophobic MD2 cavity residues, Ile63, Ile17, Phe76, Phe121, and Leu78, were principally involved in complex stability and ligand-binding affinity. In the case of MD2-morphine, most of these five residues affected ligand-binding affinity only mildly, with the exception of Phe121, which interacted directly with morphine. However, upon mutation, these residues significantly impeded the stability of the MD2-morphine complex. Considering M3G, mutation of Ile63, Phe76, and Ile117 significantly impaired complex stability and the ligand-binding affinity of MD2, and as with morphine, Leu78 and Phe121 contributed allosterically to M3G-MD2 complex stability. The results with the MD2-naloxone complex were similar to those seen with the MD2-M3G complex. The roles of residues within the MD2 pocket reportedly vary from ligand to ligand^29^, which is consistent with our observations. We were particularly interested in the effects seen on mutation of the Phe126 residue located in the gating loop. Substitution of Phe126 and Gly129 with alanine reportedly impairs LPS-induced TLR4 clustering, while having no effect on LPS binding to the TLR4/MD2 complex^30^. This suggests that MD2 ligand-binding and ligand-induced receptor clustering are two distinct phenomena in MD2-mediated TLR4 signaling. We observed similar results with computational alanine mutagenesis, wherein Phe126 mutation scarcely affected ligand-binding affinity, but considerably impaired complex stability.

These findings suggest that ligand binding and receptor clustering are two different events that occur sequentially in TLR4 signaling, and that the agonistic or antagonistic functions of ligands bound to MD2 are controlled allosterically, wherever the ligand is bound. Furthermore, the hydrophobicity of the MD2 binding pocket is pivotal for ligand recognition and binding stability, however, the more distant Phe126 loop also contributes to the agonistic or antagonistic functions of ligands.

## Discussion

A series of studies, using both *in vitro* and *in vivo* techniques, have attributed the unintended effects of opioids, such as hyperalgesia and allodynia, to opioid-induced TLR4 signaling (Table 1)^5^,^10^,^13^,^14^,^15^,^16^. Morphine and its opioid-inactive metabolite M3G are reported to activate TLR4 signaling, whereas racemic naloxone blocks this process. Our findings are consistent with recently reported biophysical and biochemical experiments, and help to clarify the mechanism of morphine-induced molecular signaling via TLR4.

MD2 has a β*-*cup fold, with clamshell-like motion of its cavity enabling it to accommodate ligands of varying sizes. Our docking results suggested that the three tested ligands bind to the pocket of MD2 with partly overlapping poses, but trigger different signaling mechanisms. Morphine robustly interacts with MD2 irrespective of TLR4 binding, although the indirect interaction of TLR4 was shown to stabilize the MD2-morphine complex (Supplementary Fig. 1). To check this effect of receptor clustering on MD2-morphine complex, we replicated the docking and MDS process (each 120 ns). However, the only variable considered during replication was ligand binding pocket and TLR4. The simulation set up and docking protocols were unchanged during replication setups. This analysis suggested that the MD2-morphine interaction and TLR4/MD2-morphine complex formation are two separate events that might occur sequentially, wherein MD2 establishes an initial interaction with morphine, before rapidly binding to TLR4 to activate downstream signaling pathways. The docking analysis of M3G with MD2 suggested that the main scaffolds of M3G and morphine initially select the same binding pocket, but that the presence of the charged glucuronide moiety of M3G was critical for the selection of the lowest energy native conformation. As with morphine, the binding of M3G to MD2 and the subsequent TLR4/MD2-M3G complex formation are likely to be separate events; however, in this case, the transition appears to be more gradual. The slower pace of complex formation is linked to the more robust interaction of MD2 with M3G than with morphine. Interestingly, the binding dynamics of naloxone with MD2 were similar to that of morphine, however, a detailed investigation of the domain dynamics of the MD2-naloxone complex revealed the mechanism by which naloxone antagonizes TLR4 (Fig. 3a).

In addition to the stability of MD2-ligand complexes, understanding the domain movements on ligand binding was a particular aim of this study. The clamshell-like motion of MD2, attributed to the β-cup folds in the MD2 mouth and the Phe126 and H1 loops, is reported to either destabilize or stabilize the heterodimeric interface of the TLR4/MD2 tetramer^31^or dimer^23^, respectively. Enhancing stability of the protein folds at the heterodimeric interface could lead to conformational change in the TLR4 transmembrane (TM) domain, and thus TLR4 oligomerization. Such changes are mainly induced by the active form of MD2, MD2^C^, and facilitated by TLR4 agonists. Both morphine and M3G maintained the active conformation of MD2, and thus confirmed their TLR4 agonistic properties. Furthermore, structural dynamics simulations with the MD2-M3G complex suggested that both Phe126 and H1 loops were important for ligand-binding affinity and the overall stability of the TLR4/MD2-M3G complex (Fig. 4). Conversely, naloxone switches the MD2 gating loop conformation from an active to an inactive form. We propose that naloxone facilitates the non-productive (inactive) conformation of MD2 in a manner similar to lipid IVa, which may then hinder the TM domain rearrangement and oligomerization of TLR4.

Our results show a correlation between the solvent accessible hydrophobic area and the cavity size of MD2, with varying degrees of cavity shrinkage observed on binding of the different ligands. This is consistent with previous work showing a correlation between the hydrophobicity of the ligand and the MD2 cavity volume^18^. When ligands were bound to MD2, the cavity size was reduced to approximately 40–50% that of the MD2-lipid IVa complex (Table 2), again suggesting the highly flexible nature of MD2 in binding TLR4-modulating molecules of varying sizes. From a structural dynamics and biomedical point of view, these findings highlight the significance of MD2 conformational plasticity in the design of molecules targeting TLR4. Furthermore, ligand binding and receptor clustering are two separate events that occur simultaneously in morphine-induced TLR4 signaling. While MD2 hydrophobicity is crucial for ligand recognition and complex stability, the binding location of ligands is less important for the allosteric control of the (ant)agonistic switching of function of the MD2-ligand complex (Fig. 7). Nevertheless, the Phe126 loop does distantly contribute to the agonistic or antagonistic properties of ligands, as was seen with the MD2-naloxone complex.

In summary, MDS provides a genuinely useful strategy for the elucidation of the properties of novel compounds, particularly those for which no high resolution structural information is known. We therefore believe that the insights generated by this study will contribute not only to our understanding of opioid-induced TLR4 signaling mechanisms, but also in the design of novel treatments for TLR4-related neuroimmune disorders.

## Methods

### Proteins structure selection and docking

Although the association between TLR4/MD2 and MD2 with the morphine, M3G, and naloxone is well established, no co-crystal structures are available to date. The structures for TLR4/MD2 (ID 3FXI^23^, co-crystalized with LPS), MD2 (ID 2E59^25^, co-crystalized with lipid IVa), and TLR4/MD2 (ID 5IJB^22^, without ligand), were retrieved from the protein data bank (PBD) and used in docking studies and as controls. These structures were checked for abnormalities and then prepared for docking simulations with morphine, M3G and naloxone. There are 3 states of MD2, based on the position of the Phe126 sidechain in the gating loop, that have been reported in crystallographic studies. In the closed state (MD2^C^) Phe126 is bent towards the hydrophobic cavity of MD2 where it binds to an agonist, in the open state (MD2°) the Phe126 sidechain is exposed to solvent and an antagonist is bound, and in the Apo state (MD2^apo^) MD2 is not bound to any ligand, and the sidechain of Phe126 is reported to be exposed to solvent^18^,^22^ (Fig. 1a). Our docking simulations were performed exclusively with the MD2^C^ state, in the presence and absence of bound TLR4. MD2°-lipid IVa, MD2^C^-LPS, and MD2^apo^ were used as controls for comparative dynamics studies with MD2^C^-naloxone, MD2^C^-morphine, and MD2^C^-M3G. MD2^apo^ was considered as a control for comparative analysis of the shrinkage in MD2 cavity, however, we did not perform detail simulation of this state as this has been previously investigated^18^.

The structures for morphine, M3G, and naloxone were retrieved from the PubChem database, prepared for docking using the MOE suite of tools, and then docked with either the MD2^C^ monomer or the heterodimeric TLR4/MD2^C^ complex. Ligands were docked with MD2^C^ using three different tools; MOE, AutoDock 4.2, and Patchdock^32^. MD2 displays one dominant hydrophobic pocket that guides the most favorable docking conformation, and all of the tools produced similar docking results with exceptionally low RMSD of the MD2-ligand complexes (Fig. 1b). Therefore, the top scoring poses for each ligand provided by MOE were selected for further analysis. A detailed description of the docking protocol has been discussed previously^33^.

### Molecular dynamics simulations (MDS)

GROMACS 5.0.7^34^, along with the recently released CHARMM36 (C36) all-atom additive protein force field (ff) module, recommended for the simulation of protein structures^35^,was used for the simulation of docked complexes and selected controls. As the ligand atoms are not recognized by the C36 ff module, the parameters for morphine, M3G, and naloxone were prepared using the SwissParam server^36^, and their charges were calculated in the MOE suite (2015.1) using the MMFF94 ff module. All simulated systems were solvated in a dodecahedron box, using the TIP3P water model, and neutralized with the addition of counter ions. Conditions mimicking a physiological salt solution were created, with Na and Cl ions being added to a concentration of 0.1 M.

The solvated systems were then energy-minimized using a steepest decent algorithm to remove unwanted steric clashes from all protein-ligand complexes. After energy minimization, the systems were grouped into protein-ligand and solvent-ions to avoid collapse, and then equilibrated for 1 ns in a constant volume (NVT). A constant temperature of 300 K was achieved using a Berendsen thermostat algorithm. All systems were equilibrated for a further 1 ns at a constant pressure (NPT) of 1 bar using a Berendsen barostat. All short-range non-bonded interactions were calculated within a cut-off of 1.2 nm, and long-range electrostatic interactions were computed using the particle mesh Ewald method with a cut-off of 1.2 nm. The LINCS algorithm was employed to constrain all bonds, and an integration time-step of 2 fs was allowed for all systems. All simulations were performed using the NPT ensemble for a total of 120 ns, and coordinates were saved at 2 fs intervals.

### Principal component analysis (PCA)

PCA is a reliable approach for unveiling high-amplitude motion in proteins that is based on the EVs of the covariance matrix of atomic fluctuations^37^. The GROMACS gmx-sham module was utilized to calculate FELs. cPCA and dPCA were performed using the Cartesian coordinates of all MD2 protein backbone atoms, and the backbone dihedral angles of the MD2 Phe126 loop, respectively. In cPCA, the first ten EVs (of MD2-ligand complexes) represented approximately 80% of the fluctuation, and the first two EVs, corresponding to PC1 and PC2, were aligned to investigate the FEL and to calculate the Gibbs free energy. In dPCA, the dihedral angles (φ and ψ) of the Phe126 loop backbone atoms were extracted and investigated. The plateaus of the FEL were calculated and displayed using a trial version of Mathematica.

### Alanine scanning and relative affinity and stability

Mutational studies are commonly used to investigate the contribution of a particular amino acid residue to ligand-binding affinity or complex stability. The latest versions of the MOE suite (versions 2013 onwards) include protein design tools that can predict the contribution of each amino acid to complex stability, and their affinities for the bound ligand. The methods used in this study were as described previously^33^, with the following exceptions:, the ‘mutation search’ method from the Amber10:EHT forcefield module^38^ was used instead of MMFF94x. The LowMode MD method generates mutations before applying MDS at a constant temperature for a short time^39^ and minimizing the energy of resulting mutant complexes under the forcefield. Finally, each mutant state is assigned a relative score relating to the change in stability and ligand-binding affinity of the mutant complex.

### Data analysis and graphics

Most of the data were analyzed with built-in modules of GROMACS v5.0.7. Graphical images were produced with PyMol^40^ and VMD^41^. Interface analyses were performed in MOE, VMD, and UCSF Chimera, and images were generated in MOE and Chimera. All computational studies were performed on a Dell PowerEdge server with a CentOS6 GNU/Linux operating system.

## Additional Information

**How to cite this article:** Shah, M. *et al*. A structural insight into the negative effects of opioids in analgesia by modulating the TLR4 signaling: An *in silico* approach. *Sci. Rep.* **6**, 39271; doi: 10.1038/srep39271 (2016).

**Publisher's note:** Springer Nature remains neutral with regard to jurisdictional claims in published maps and institutional affiliations.

## Supplementary Material

Supplementary Information

## Figures and Tables

**Table 1:** Interactions of ligands with TLR4/MD2, investigated with *in vitro, in vivo* and *in silico* experiments.

	Cell line	Animal	*In silico*		TLR4/MD2	Binding location		Assay	Ref
			Docking	MDS		TLR4	MD2		
**Morphine**	RAW264.7, HEK293-hTLR4	Mice	AutoDock	ND	LPS pocket	NS	Val48, Ile63, Phe76, Phe147,	ND	14
	Endothelial, HEK293-hTLR4	Rat	PyRx	ND	LPS pocket	Yes	LPS pocket	ND	5
	HEK Blue- hTLR4	ND	Placement	CHARMM (3 ns)	NS	NS	Arg90, Glu92, Lys122, Phe126, Ser127	yes	15
	—	Rat/Mice	PyRx	ND	LPS pocket	NS	LPS pocket	yes	16
**M3G**	RAW264.7,HEK293-hTLR4	Mice	AutoDock	ND	Suggested	NS	Val48, Phe76, Ile63, Phe147	ND	14
	Endothelial	Rat	PyRx	ND	Suggested	Yes	LPS pocket	yes	5
	HEK 293,BV-2 cells	Rat	AutoDock	ND	LPS pocket	NS	LPS pocket	yes	10
**Naloxone**	RAW264.7,HEK293-hTLR4	Mice	AutoDock	ND	Yes	NS	Val48, Ile63, Phe76, Phe147	ND	14
	HEK293,	Rat	AutoDock	ND	LPS pocket		LPS pocket	yes	10
	HEK293,Microglial cell	ND	ND	ND	NS	NS	NS	yes	13
	—	Rat/Mice	PyRx	ND	LPS pocket	NS	LPS pocket	yes	16

LPS: Lipopolysaccharide; ND: Not done; NS: Not suggested; ns: Nano second; Ref: References.

**Table 2:** Summary of the docking results and interface analysis for ligand-bound MD2.

Comp	Ligand	Stability	Area (Hyd, A^2^)	Sol (kcal/mol)	Aff (kcal/mol)	H/Ar-bond	Hyd-bond
**Analysis of the stability of MD2- or TLR4/MD2-bound opioids and their initial conformational, and of the hydrophobic pocket interface**							
MD2	Morph(A)	No	881 (11.8%)	−27.33	−6.32	Phe151 (Ar)	Ile32,52,153; Leu61; Phe121,151
	Morph(B)	No	884 (11.8%)	−31.26	−7.85	Ile63, (Ar)Phe104 (Ar)	Ile44,63,117; Phe76,104,147; Leu149
	M3G(A)	No	840 (11.5%)	−34.70	−7.60	—	Ile32,52,153; Leu78; Phe151
	M3G(B)	Yes	870 (11.6%)	−38.17	−8.6	Phe76 (Ar)	Ile46,63,94,117; Leu61,78; Val135; Phe104,151
	Nlx(B)	No	873 (11.6%)	−34.60	−8.22	Phe104 (Ar)	Ile63,94,117; Leu71;Val113; Phe76,104,147
TLR4/MD2	Morph(A)	Yes	873 (11.6%)	−21.24	−5.44	Phe76 (Ar)	Phe76; Leu78; Ile94,117
	M3G(A)	No	850 (11.5%)	−35.10	−7.70	Tyr102 (H), Phe151 (Ar)	Ile63; Leu61,78; Phe119,151; Val135
	Nlx(A)	Yes	872 (11.6%)	−27.52	−6.64	Phe151 (Ar)	Ile32,46,63; Leu61; Val24,48,135; Phe151
**Investigation of the changes in pocket area and ligand and interfacial residue conformation**, **after 120 ns in stable complexes**							
MD2	M3G(B)	Yes	524 (6.6%) (5.1%↓)	−46.13	−9.07	Glu92 (H), Ser103 (H)	Ile94,117; Leu61,78; Val135; Phe76, 119
TLR4/MD2	Morph(A)	Yes	480 (7.0%) (4.0%↓)	−31.40	−7.20	Thr81 (H), Phe121 (Ar)	Val82; Leu87; Ile80,124,153; Phe76,119,121
	Nlx(A)	Yes	523 (7.4%) (4.2%↓)	−33.18	−6.62	Glu92 (H), Ser118 H),	Ile63,117; Leu61,78; Phe76

↓: Decrease in area; Hyd: Hydrophobic; Sol: Solvation energy; Aff: Affinity; Nlx: Naloxone; Ar: Arene bonds.
