# Supplementary material for: A structural insight into the negative effects of opioids in analgesia by modulating the TLR4 signaling: An in silico approach
Source: Sci Rep. 2016 Dec 16;6:39271. doi: 10.1038/srep39271 (PMC5159903; doi:10.1038/srep39271)
Supplement: Supplementary Information [file srep39271-s1.pdf]

<Supplementary Information>

A structural insight into the negative effects of opioids in analgesia by modulating the TLR4 signaling: An *in silico* approach

Masaud Shah[1], Muhammad Ayaz Anwar[1], Dhanusha Yesudhas[1], JayalakshmiKrishnan[2], Sangdun Choi[1]*

[1]Department of Molecular Science and Technology, Ajou University, Suwon, 443-749, Korea
[2]Department of Life Sciences, Central University of Tamil Nadu, Neelakudi, Thiruvarur, Tamil Nadu, India

Masaud Shah: masaudghalib@hotmail.com
Muhammad Ayaz Anwar: ayaz@ajou.ac.kr
Dhanusha Yesudhas: dhanusha2504@gmail.com
JayalakshmiKrishnan: jayalakshmi@cutn.ac.in

*Corresponding author:
Sangdun Choi
Department of Molecular Science and Technology, Ajou University, Suwon, 443-749, Korea
Email: sangdunchoi@ajou.ac.kr

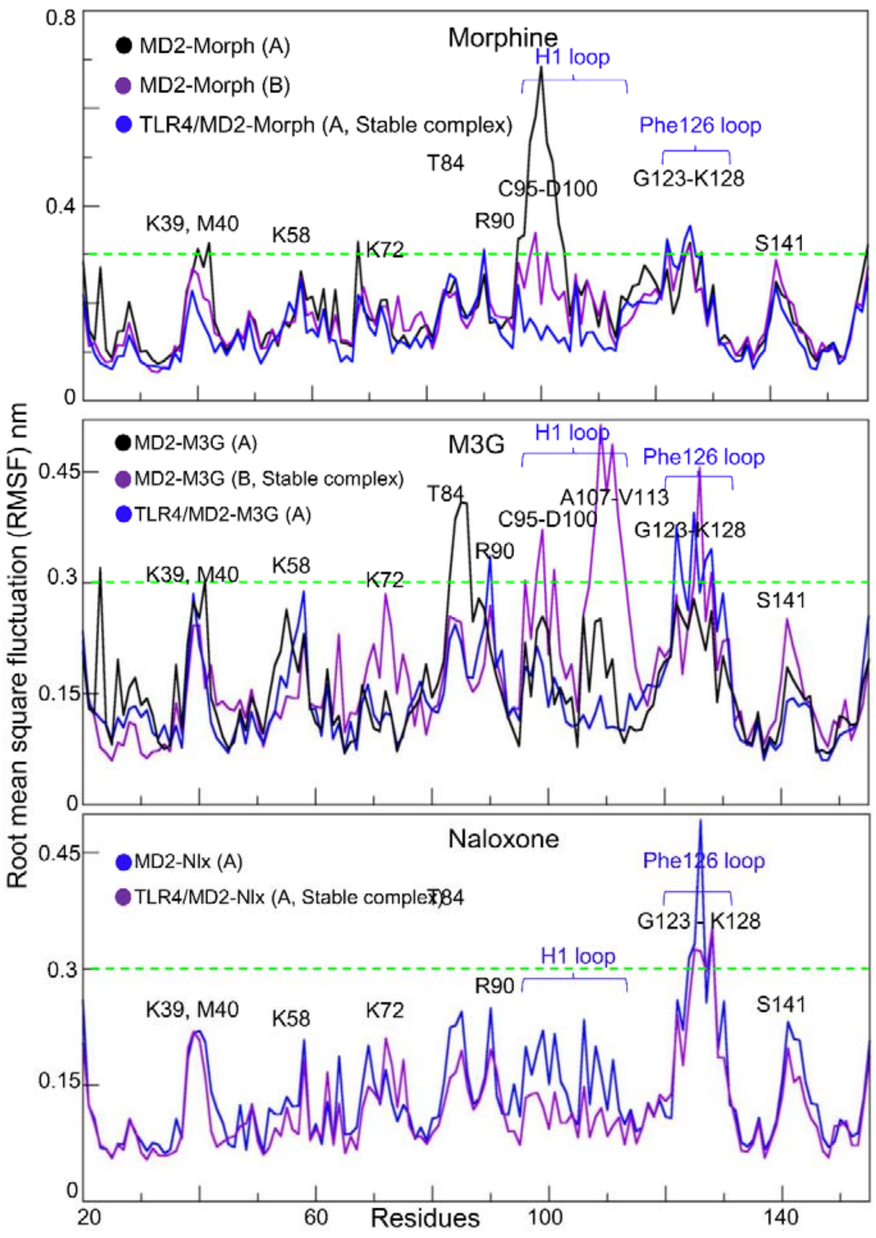

Supplementary Figure 1. Comparative root-mean-square fluctuation (RMSF) in MD2 residues and the effect of TLR4. In the absence of TLR4, residues Cys95 to Ser102 exhibit high fluctuation. These residues, along with others, are stabilized in the TLR4/MD2-morphine complex. TLR4 plays a limited role in MD2-M3G complex stability, and a reduction in loop fluctuation can be seen in the RMSF plot. Additionally, TLR4 facilitated the formation of MD2 complexes with both morphine and naloxone. A and B represent the cavities in which the ligand was docked. Morph; morphine; Nlx: naloxone.

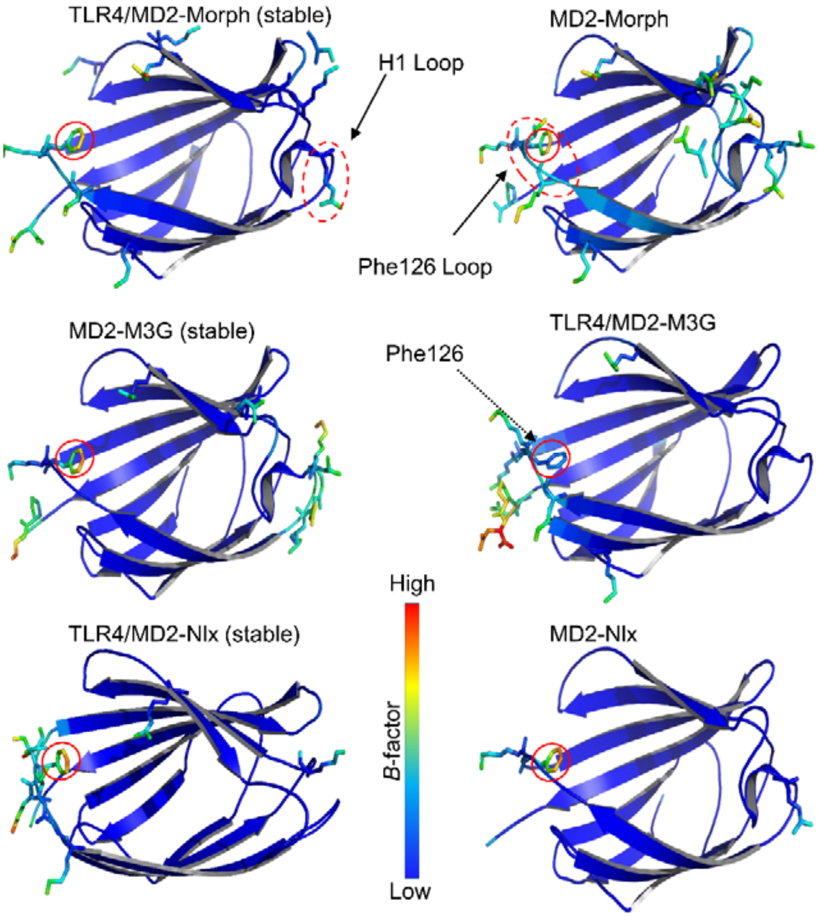

Supplementary Figure 2. The effect of TLR4 on MD2 stability as determined by β-factors. Interaction with TLR4 greatly reduces the vibrations of the flexible loop regions of MD2. However, β-factors of various degrees are seen in the Phe126 loop. In the presence of the TLR4 agonists morphine and M3G, the gating loop residue Phe126 exhibits only minimal vibrations. However, in the presence of naloxone, the entire gating loop vibrates considerably, reflecting the antagonistic nature of this ligand. Morph; morphine; Nlx: naloxone.

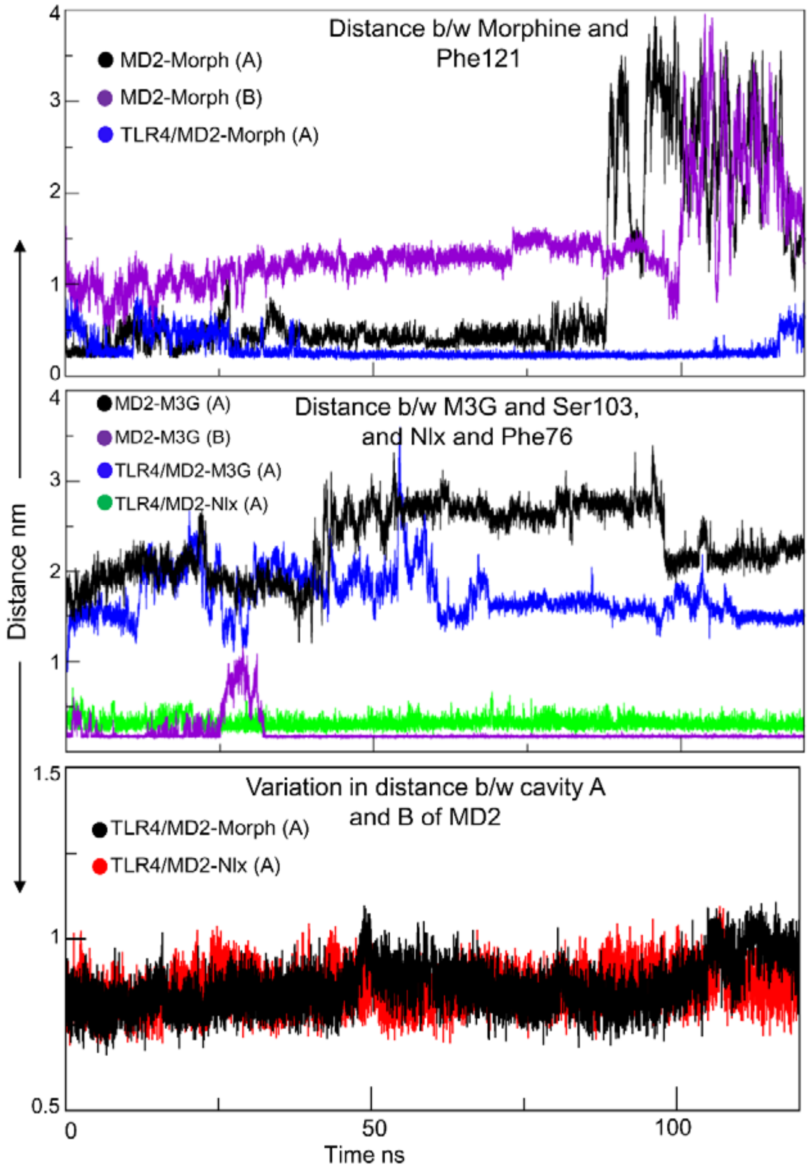

Supplementary Figure 3. Variation over time of the distance between ligands and interacting residues. The distance between Phe121 and morphine was measured over time to track the relative position of the ligand in the MD2 cavity. In the presence of TLR4, morphine remains bound to MD2 via the Phe121 residue, however, the MD2-morphine complex dissociates after time in the absence of TLR4. Similarly, the distance between Phe76 and naloxone was monitored, and this ligand remained bound to MD2 in the presence of TLR4. Unlike with morphine and naloxone, the stability of the MD2-M3G complex does not require TLR4 binding, and is instead cavity- and pose-dependent. M3G interacts

strongly with Ser103 of cavity B, but dissociates from MD2 if docked within cavity A, regardless of TLR4 binding status.

The bottom graph represent the change in the distance between cavity A and B. The distance was measured as a center of mass (COM) of the cavity A residues (Ile80, Val82, Phe121, and Tyr131) and cavity B residues (Tyr65, Phe76 and Phe104). As can be seen there is no change in the distance between these two cavities; this support the clamshell like motion of MD2, which can shrink or open MD2 cavity from top and bottom but do not from lateral sides. A and B denote the cavities in which the ligand was docked. Morph; morphine; Nlx: naloxone.

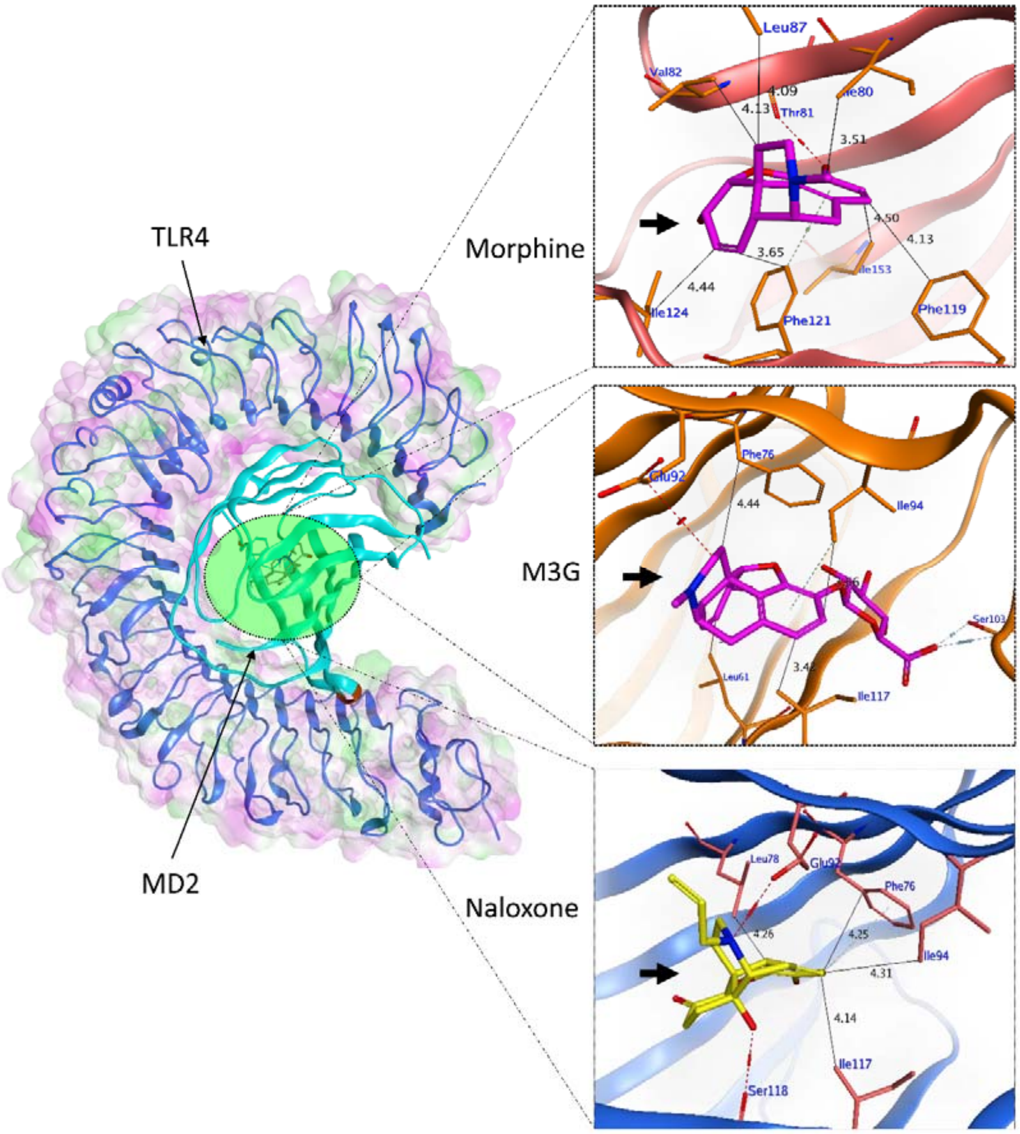

Supplementary Figure 4. Pictorial representation of the bound morphine, M3G and naloxone in MD2 cavity in TLR4/MD2 complex. TLR4 in TLR4/MD2 complex influence the stability of morphine and naloxone in MD2 cavity and has less effect on the MD2-M3G complex. The close-up pictures represent the stable conformation of each ligand. However, M3G shown in the clos-up picture is taken from MD2-M3G complex, which was not bound to TLR4.

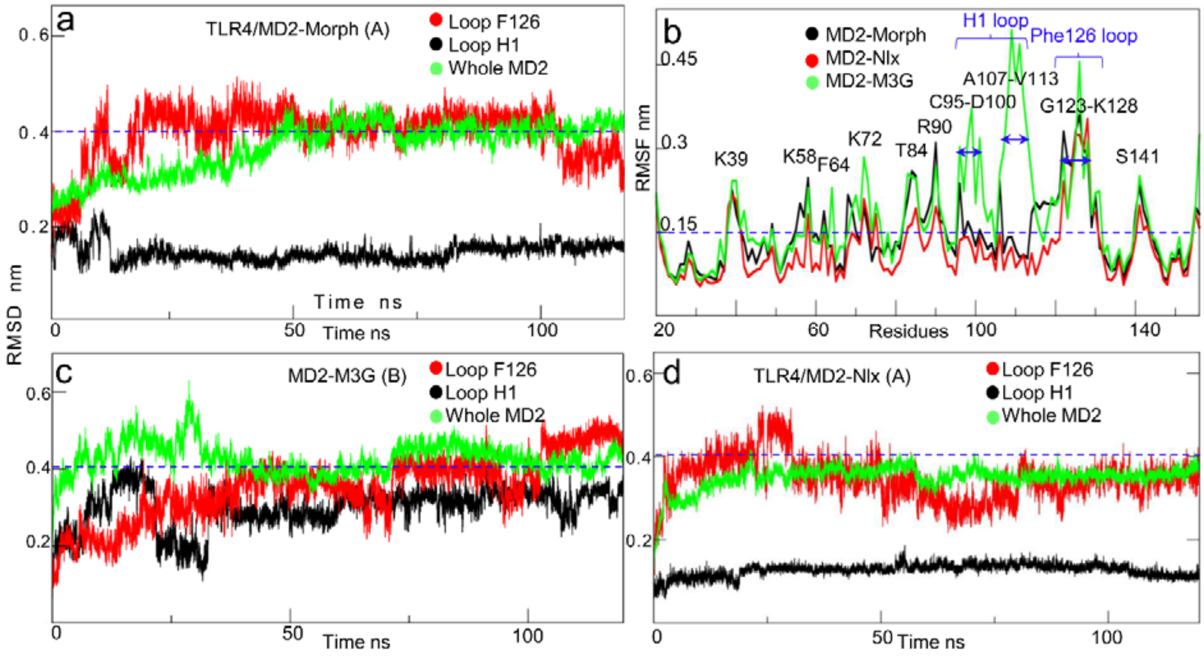

Supplementary Figure 5. Comparative dynamics of whole MD2 and its Phe126 and H1 loops. (**a**) Morphine interacts with MD2 near to the gating loop to induce receptor clustering. The H1 loop remains stable in the MD2-morphine complex, suggesting a limited role in ligand binding. (**b**) Root mean square fluctuation (RMSF) was observed in the ligand-binding amino acid residues of MD2, and while all of the ligands elicited gating loop fluctuation, only naloxone switched the MD2 conformation from the productive ($MD2^C$) to the non-productive ($MD2^O$) state. High fluctuations were also observed in the MD2 H1 loop on M3G binding. (**c**) Both the Phe126 and H1 loops exhibited considerable fluctuation in the MD2-M3G complex, indicating that both have roles in ligand binding and receptor clustering. (**d**) Although MD2 complexes with morphine or naloxone displayed similar structural dynamics and residue fluctuation, they ultimately provoked opposite outcomes; while morphine facilitated the active $MD2^C$ state, naloxone promotes the inactive $MD2^O$ state. A and B indicate the cavities in which the ligand was docked. Morph; morphine; Nlx: naloxone.
